# Duration of the Humoral Immune Response to Influenza Vaccination in a Cohort of Repeatedly Vaccinated Adults over Four Consecutive Epidemic Seasons: 2018/19–2021/22 Observational Study

**DOI:** 10.3390/vaccines14090800

**Published:** 2026-09-11

**Authors:** Mariia V. Sergeeva, Polina Kudar, Yulia Desheva, Ekaterina Romanovskaya-Romanko, Vera Krivitskaya, Janna Buzitskaya, Marina A. Stukova, Irina Kiseleva, Dmitry Lioznov

**Affiliations:** 1 Smorodintsev Research Institute of Influenza, Ministry of Health of the Russian Federation, Saint Petersburg 197022, Russiamarina.stukova@influenza.spb.ru (M.A.S.);; 2 FSBSI ‘Institute of Experimental Medicine’, Saint Petersburg 197022, Russiairina.v.kiseleva@mail.ru (I.K.)

**Keywords:** influenza vaccination, repeated vaccination, humoral immunity, antibody persistence, hemagglutination inhibition, neuraminidase inhibition, seroprotection

## Abstract

**Background/Objectives**: The impact of repeated annual influenza vaccination on long-term immunogenicity and protection remains debated. Although reduced fold-increases in antibody titers are often observed in repeatedly vaccinated individuals, the effect on the duration of the humoral response remains poorly understood. This study evaluated the magnitude and persistence of the humoral immune response over four consecutive seasons. **Methods**: An open-label observational study was conducted between 2018 and 2022 and involved 28 healthy adults (median age, 55 years) immunized with seasonal inactivated influenza vaccines. Hemagglutination inhibition (HAI) titers were assessed at five time points annually. Secondary endpoints included neutralizing (MNA) and neuraminidase-inhibiting (ELLA) antibody responses measured at baseline and three weeks after vaccination. The duration of antibody responses above the seroprotection threshold (titer ≥ 40) was compared across seasons using the Mantel–Cox test. **Results**: Antibody dynamics differed by vaccine component. The highest GMT and fold-increase were observed for A/H1N1pdm09 in 2018/19, followed by a significant decline after the second vaccination, that was more pronounced for neutralizing antibodies. The response to N1 neuraminidase remained weak throughout the study. For A/H3N2, baseline HAI GMTs increased significantly after each subsequent vaccination, whereas N2 neuraminidase responses peaked after the first dose and declined thereafter. Influenza B responses were weaker than those to influenza A. A significant increase in B/Yamagata titers in 2020/21 was attributed to the introduction of quadrivalent vaccines. A/H1N1pdm09 HAI responses were long-lasting, with titers remaining at or above the seroprotection threshold for one year. The duration of the A/H3N2 antibody response increased significantly over four seasons, likely because of annual strain updates. A similar trend was observed for B/Victoria after strain changes. **Conclusions**: Repeated annual influenza vaccination did not negatively affect post-vaccination antibody titers to the HA vaccine antigens in the studied cohort. Regular updates to vaccine strains may enhance the persistence of HA-targeted humoral immune responses.

## 1. Introduction

Annual influenza vaccination is the primary strategy for mitigating the global burden of seasonal influenza epidemics [1]. The World Health Organization (WHO) continuously updates the recommended strains to match rapidly evolving circulating viruses. However, the impact of regular booster immunizations on long-term protection and overall vaccine efficacy remains a subject of active scientific debate. The protective value of each subsequent vaccination may be reduced in repeatedly vaccinated individuals [2,3,4]. Individuals vaccinated annually often experience a weaker fold increase in antibody titers than those vaccinated for the first time [5]; however, repeated vaccination maintains high seroprotection rates from year to year [6]. Prior vaccination may reduce antibody affinity maturation to the hemagglutinin (HA) head of the current vaccine strain [7]. When individuals are exposed to a newly updated vaccine strain, circulating cross-reactive memory B cells may dominate the response, potentially limiting the *de novo* production of highly specific antibodies against novel epitopes on the updated HA [8,9].

Furthermore, repeated annual vaccination produces markedly different immune dynamics across influenza virus subtypes [5]. For A/H1N1pdm09, successive immunizations often lead to a progressive reduction in the post-vaccination fold increase, driven by the rapid clearance of vaccine antigens by high baseline levels of neutralizing antibodies. Conversely, A/H3N2, which is characterized by substantial antigenic drift, often exhibits a cumulative increase in binding antibodies across multiple seasons; however, this quantitative increase does not always translate into enhanced functional neutralization [10,11].

In recent years, the role of neuraminidase (NA) as an additional target of antiviral immunity has attracted considerable attention. Anti-NA antibodies are recognized as an independent correlate of protection: they inhibit enzymatic activity, block virion release, exert Fc-dependent effector functions and limit viral transmission [12,13,14]. Vaccination has been shown to induce a significant and durable antibody response to NA, with titers remaining elevated for up to six months [15]. Nevertheless, anti-NA antibodies are not included in routine immunogenicity assessments [16], and the dynamics of NA-inhibiting antibodies after repeated vaccinations remain poorly understood, particularly with respect to long-term persistence and cross-protective immunity.

Although immediate post-vaccination geometric mean titers (GMTs) are important, the duration of protection throughout the epidemic season (intra-seasonal waning) remains understudied. Several studies indicate that protection may wane as early as five months after vaccination [17]. Influenza vaccine effectiveness declines by approximately 6–11% per month after vaccination, potentially leaving individuals vulnerable during late-season outbreaks [18,19]. The rate of decline may differ substantially among influenza subtypes. Influenza A/H1N1pdm09 tends to induce more stable, long-lasting antibody titers, whereas influenza A/H3N2 is characterized by more rapid waning of immunity [20]. At the same time, the influenza B lineages (Victoria and Yamagata) often show lower overall immunogenicity and distinct persistence patterns depending on the vaccine formulation (trivalent vs. quadrivalent) [21].

Long-term, multi-year observational cohorts that track antibody persistence across multiple consecutive seasons are rare. Although influenza antibody waning has been examined over periods of approximately 12–18 months [22,23,24], longitudinal cohorts involving repeated serological assessments of the same individuals across three or more consecutive influenza seasons remain limited [25]. Furthermore, most studies prioritize HAI assays, leaving the dynamics of functional neutralizing and neuraminidase-inhibiting antibodies insufficiently characterized. To address these gaps, this prospective four-year observational study (2018–2022) evaluated the magnitude, breadth, and long-term persistence of the humoral immune response in repeatedly vaccinated adults. We assessed the dynamics of virus HA- and NA-specific antibodies across four consecutive epidemic seasons to determine how regular vaccine strain updates and cumulative vaccination history modulated long-term humoral immune memory.

## 2. Materials and Methods

### 2.1. Study Design and Participant Cohort

This open-label, prospective observational cohort study was conducted over four consecutive influenza epidemic seasons between 2018 and 2022. Each season, adults with no contraindications to influenza vaccination were enrolled. Eligible participants met the following inclusion criteria: age ≥ 18 years at enrollment; no known history of immunodeficiency or use of immunosuppressive therapy; commitment to receiving the annual seasonal influenza vaccine; and compliance with the multi-year blood sampling schedule. Participants who completed four years of observation, who received annual vaccination and donated blood samples at the scheduled time points, were included in the final study cohort. Detailed individual reasons for attrition were not assessed.

Participants were monitored for influenza and other respiratory viral infections during follow-up. In cases of acute respiratory illness, nasopharyngeal swab samples were tested using a commercial qRT-PCR-based assay for influenza viruses, SARS-CoV-2, and a panel of common respiratory viruses (AmpliSens; FBIS Central Research Institute of Epidemiology of Rospotrebnadzor, Moscow, Russia).

The study protocol was approved by the local institutional ethics committee, and all participants provided written informed consent before any study-related procedures were performed.

### 2.2. Vaccines and Vaccination Schedule

Participants were immunized annually during the autumn months (September to November) of four consecutive seasons: 2018/19, 2019/20, 2020/21, and 2021/22. The administered vaccines were commercially available seasonal inactivated influenza vaccines licensed in the Russian Federation, including split-virion and adjuvanted subunit formulations. Vaccine strains were updated annually in accordance with World Health Organization (WHO) recommendations for the Northern Hemisphere (Table 1).

### 2.3. Blood Sampling Timeline

To monitor the development, peak, and long-term persistence of the humoral immune response, peripheral blood samples were collected from each participant at five time points annually. The first sample (V1) was obtained immediately before vaccination on day 0, and served as the baseline. A second sample (V2) was collected three weeks (21 days) after immunization to capture the peak post-vaccination response. To assess the durability of the antibody response during the epidemic season, additional samples were collected approximately three months (V3) and six months (V4) after vaccination. Finally, a fifth sample (V5) was obtained 12 months after vaccination, immediately before administration of the subsequent season’s vaccine, to evaluate pre-season antibody titers and long-term persistence.

All blood samples were centrifuged at 1500× *g* for 10 min to obtain serum. The serum was then aliquoted and stored at ≤−20 °C until serological testing. Each aliquot was thawed only once.

### 2.4. Serological Assays

#### 2.4.1. Hemagglutination Inhibition (HAI) Assay

Total strain-specific antibodies targeting the influenza hemagglutinin head were quantified using a standard HAI assay. Serum samples were treated with receptor-destroying enzyme (RDE) from Vibrio cholerae to eliminate non-specific inhibitors and were then adsorbed with chicken red blood cells to remove non-specific agglutinins. Serial two-fold serum dilutions were incubated with 4 hemagglutinating units (HAU) of the respective vaccine virus strains before the erythrocyte suspension was added. The HAI titer was defined as the reciprocal of the highest serum dilution that completely inhibited hemagglutination.

#### 2.4.2. Microneutralization Assay (MNA)

Functional neutralizing antibody responses were assessed as a secondary endpoint using an in vitro microneutralization assay. Briefly, RDE-treated, heat-inactivated serum samples were serially diluted and incubated with 100 TCID_50_ (50% tissue culture infectious dose) of the corresponding influenza vaccine virus strains. The virus-serum mixture was transferred to confluent Madin-Darby canine kidney (MDCK) cell monolayers. After incubation, viral replication was determined using cell-based enzyme-linked immunosorbent assay (ELISA) targeting viral nucleoprotein. MDCK cells (London line) were obtained from the International Reagent Resource (IRR; formerly the Influenza Reagent Resource; Catalog no. FR-58).

#### 2.4.3. Enzyme-Linked Lectin Assay (ELLA)

In this assay, 96-well, flat-bottom ELISA plates with a high protein-binding capacity were coated with 150 μL of fetuin solution at a concentration of 50 μg/mL, in 0.1 M carbonate-bicarbonate buffer (pH 9.5–9.7). The plates were incubated overnight at 4 °C to allow substrate adsorption. Before use, the coated plates were washed twice with sterile phosphate-buffered saline (PBS, pH 7.4) to remove unbound fetuin. Working dilutions of the diagnostic reassortant viruses A/H6N1, A/H6N2 and A/H2NB (described previously in [26,27]) were freshly prepared in 0.01 M PBS supplemented with 10 mg/mL bovine serum albumin (BSA). Next, 65 μL of the virus working dilution was added to each well containing serially diluted serum samples. For assay validation, each plate included both negative control wells (containing 130 μL of PBS with 10 mg/mL BSA) and positive control wells (containing 65 μL of virus working dilution and an equal volume of PBS with 10 mg/mL BSA). After incubation, residual neuraminidase activity was measured by adding TMB as a chromogenic substrate, and optical density (OD) was recorded at 450 nm. Serum antibody titers were defined as the reciprocal of the highest dilution resulting in 50% inhibition of neuraminidase activity.

Because of methodological and reagent limitations: NAI responses were evaluated only at baseline (V1) and three weeks after vaccination (V2); the NA antigens used in the assays were the same in all four years and were not identical to the corresponding vaccine strains; influenza B NAI assays were constrained by the use of the H2NB diagnostic platform; individuals with pre-existing H2-specific antibodies, that could have interfered with the assay, were excluded from the calculations.

#### 2.4.4. Antigens

The viruses used as antigens in the HAI and MNAs corresponded to the vaccine strains included in the respective seasonal vaccine formulations (Table 1). Vaccine virus strains were obtained from the WHO Collaborating Centers for Reference and Research on Influenza through the GISRS program for the National Influenza Centre at the Smorodintsev Research Institute of Influenza (St. Petersburg, Russian Federation).

The diagnostic reassortant viruses A/H6N1, A/H6N2, and A/H2NB used in ELLA have been described previously [26,27]. The first two strains bear HA from the A/herring gull/Sarma/51c/2006 (H6N1) influenza virus and NA from the A/South Africa/3626/13 (H1N1)pdm09 or A/Hong Kong/4801/2014 (H3N2) strains. The latter two strains bear HA from the A/Leningrad/134/17/57 (H2N2) influenza virus and NA from the B/Brisbane/60/2008 (B/Victoria) or B/Phuket/3073/2013 (B/Yamagata) strains. Viruses were obtained from the collection of the Virology Department, Institute of Experimental Medicine (St. Petersburg, Russian Federation).

#### 2.4.5. Assay Setup Conditions

The HAI and MNAs were performed contemporaneously, and multiple samples from each participant (all time points within one season) were tested in parallel. Control serum samples were used to monitor inter-assay variability when samples were tested against the same antigen in different seasons. The ELLA was performed retrospectively, and all samples from each participant were tested in parallel.

### 2.5. Statistical Analysis

Statistical analyses were conducted using Prism 10.4.1 (GraphPad, Boston, MA, USA) or RStudio v. 2024.04.2+764 (Posit Software, Boston, MA, USA).

Geometric mean titers (GMTs) and 95% confidence intervals (CIs) were calculated for the HAI, MNA, and ELLA results at each designated time point. Antibody titers below the assay detection limit (<10) were assigned a conventional value of 5 for computational purposes [28]. Antibody fold-increases were calculated as the ratio of the post-vaccination titer at V2 (day 21) to baseline titer at V1 (day 1). Differences in these metrics across seasons were assessed using mixed-effects model (REML) applied to log-transformed values. The Geisser-Greenhouse correction was used, because sphericity could not be assumed, and data were not imputated at this stage. Given the observational study design, a less stringent post-hoc analysis was performed using Holm–Sidak multiple comparison tests. Statistical tests were performed independently for each vaccine component (A/H1N1pdm09, A/H3N2, B/Victoria, and B/Yamagata). The family-wise alpha threshold was set at 0.05. Differences with *p* < 0.05 were considered statistically significant.

Seroprotection was defined as an HAI titer ≥ 40. The seroprotection rate was defined as the percentage of participants with an antibody titer ≥ 40. The duration of the immune response after immunization, defined as the persistence of antibody titers at or above the seroprotection threshold was analyzed and plotted using Kaplan–Meier survival curves. Differences in the duration of antibody persistence across seasons was evaluated using the Mantel–Cox (log-rank) test.

## 3. Results

During four consecutive influenza epidemic seasons between October 2018 and November 2022, we conducted a longitudinal study of the humoral immune response in vaccinated adults. Annual vaccinations were administered from September to November, beginning in October 2018 and ending in November 2021; follow-up sampling continued until November 2022. The cohort comprised 28 participants ≥ 18 years old who received inactivated influenza vaccines annually and donated blood samples at the scheduled time points (at least four time points per year; follow-up rates are presented in Supplementary Figure S5). The cohort was predominantly female (75%) and White (100%), and the median age was 55 years (Table 2). Several participants belonged to risk groups because of chronic respiratory diseases; the prevalence of these conditions did not exceed that in the general population [29,30]. Chronic cardiovascular disease, mainly hypertension, was present in 32% of participants, approximating the prevalence in the general population of a similar age [31]. More than half of the cohort had a history of influenza vaccination, and 39% had been vaccinated in the 2017/18 season immediately preceding the study. During the study participants received seasonal influenza vaccines, licensed and approved for use in adults, including those aged ≥60 years. The proportion of quadrivalent formulations increased from 0% in the first year to 82% in the fourth year as quadrivalent influenza vaccines were gradually introduced in the Russian Federation (Table 3). The increased use of split vaccines in the fourth study year was most likely driven by participants’ greater preference for quadrivalent formulations. Because the study was open-label, participants were allowed to choose which vaccine they received. During the 2021/2022 season, all quadrivalent influenza vaccines available in the Russian Federation were split-virus formulations. Influenza epidemiology differed among seasons. In 2018/19, A/H1N1pdm09 and A/H3N2 viruses co-circulated. In 2019/20, A/H1N1pdm09 predominated during the first half of the season, followed by B/Victoria-lineage viruses near the end of the season. In 2020/21, only a few cases of B/Victoria infection were recorded. In 2021/22, the epidemic was caused by an A/H3N2 virus that was antigenically distinct from the vaccine strain (Table A1) [32,33]. No cases of symptomatic influenza were recorded in the vaccinated cohort during the four-year observation period. One case of asymptomatic influenza B infection in the 2020/21 season was suspected on the basis of serological data (Table A1). We therefore considered it unlikely that circulating viruses substantially affected either the magnitude or the duration of the influenza-specific immune response in the study cohort.

**Figure 1:**
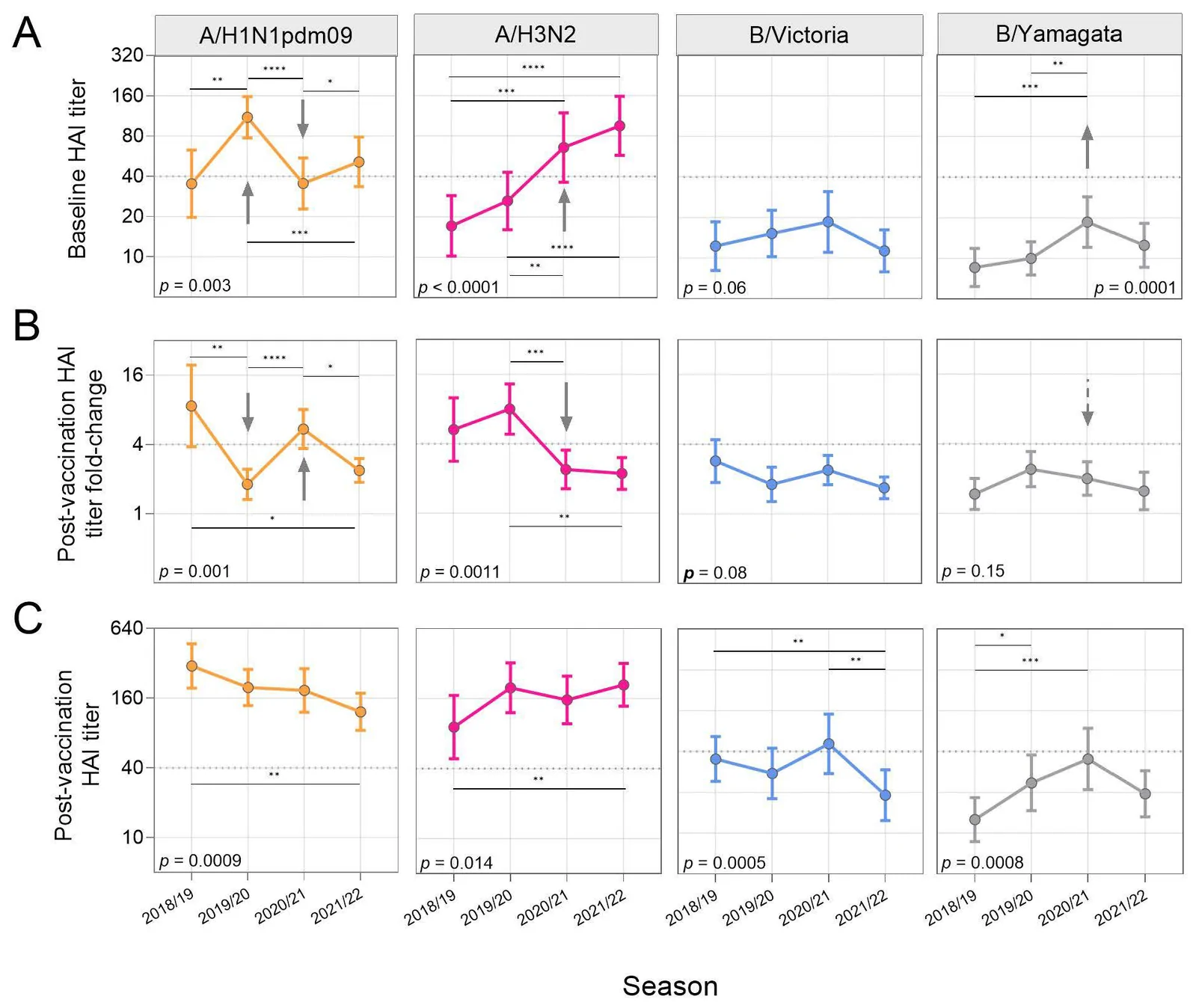
Pre- and post-vaccination hemagglutination-inhibiting antibody titers during four influenza epidemic seasons. (**A**) Pre-vaccination baseline antibody titers measured on day 1; (**B**) fold changes in post-vaccination antibody titers measured on day 21 relative to baseline (day 1); and (**C**) post-vaccination antibody titers measured on day 21. GMTs with 95% CIs are shown. Antibodies were measured against the corresponding vaccine strains. Results of the mixed-effects model are shown in the lower-right corner of each graph (*p* values for the season factor). Statistically significant differences between seasons are indicated by asterisks: * *p* < 0.05, ** *p* < 0.01, *** *p* < 0.001 and **** *p* < 0.0001, based on Holm–Sidak multiple-comparison tests. The dotted line indicates the seroprotection threshold in (**A**,**C**) and the biologically significant titer fold increase in (**B**). Individual data are presented in Supplementary Figure S1.

Distinct four-year trends in pre-vaccination baseline antibody titers were observed for the individual vaccine components, with an overall classic inverse relationship between baseline titers and fold-changes (Figure 1A). Baseline antibody titers to the A/H1N1pdm09 component showed statistically significant inter-seasonal fluctuations (up to a 3.1-fold difference). Vaccination in the first season (2018/19) produced the greatest increase in antibody titers (an approximately 10-fold increase in GMT, from 35 to 338). The high titers of A/H1N1pdm09-specific antibodies induced by the first vaccination persisted for one year and resulted in a high baseline value during the second year (GMT = 189), leading to a significant 5.7-fold reduction in the antibody fold increase (Figure 1B, downward gray arrow). Conversely, during the third year (2020/21), a decrease in baseline antibody titers (GMT = 35) allowed a significant 3-fold increase in antibody fold-changes after vaccination. In the fourth year, the A/H1N1pdm09-specific baseline GMT stabilized near the seroprotection threshold and only a modest fold-increase was observed. The post-vaccination GMT decreased progressively and was significantly lower in the 2021/22 season (a 2.5-fold reduction relative to 2018/19). Nevertheless, the post-vaccination GMT remained above the seroprotection threshold (GMT = 124).

A pronounced trend toward the accumulation of post-vaccination immunity to the A/H3N2 component was observed (Figure 1A,C). With each subsequent season, pre-vaccination antibody titers increased significantly and consistently (*p* < 0.0001), from GMT of 17 to a maximum GMT of 95 by the fourth year. A classic inverse relationship between baseline titers and post-vaccination fold increases was observed only during the third year (gray arrows), when the baseline GMT exceeded 80. Beginning in the first season, the post-vaccination GMT rose above the seroprotection threshold, and a significant increase was observed in the fourth season (GMT = 223).

Baseline antibody titers and fold changes for the B/Victoria-component showed the most stable and consistent pattern over the four years (Figure 1, blue line). No statistically significant differences were found between seasons. A modest upward trend in baseline titers (approximately 1.6-fold) was observed during the third year. Post-vaccination GMTs for influenza B/Victoria (maximum GMT = 50) were lower than those for both influenza A components, and a significant 2.3-fold reduction was observed in the fourth season.

For the B/Yamagata component, baseline HAI antibody titers were consistently low during the first two years (Figure 1A, gray line). However, in the third year (2020/21), a statistically significant 2.1-fold increase in pre-vaccination titers was recorded (*p* = 0.03), most likely because of the transition to quadrivalent vaccines. Post-vaccination titers increased significantly during the 2019/20 and 2020/21 seasons (a 2.9-fold difference relative to 2018/19). Differences in antibody fold changes across years did not reach statistical significance (*p* = 0.064), and remained consistently small throughout the study. The strongest responses to both influenza B-components were observed during the third year, when GMTs reached the seroprotection threshold.

**Figure A1:**
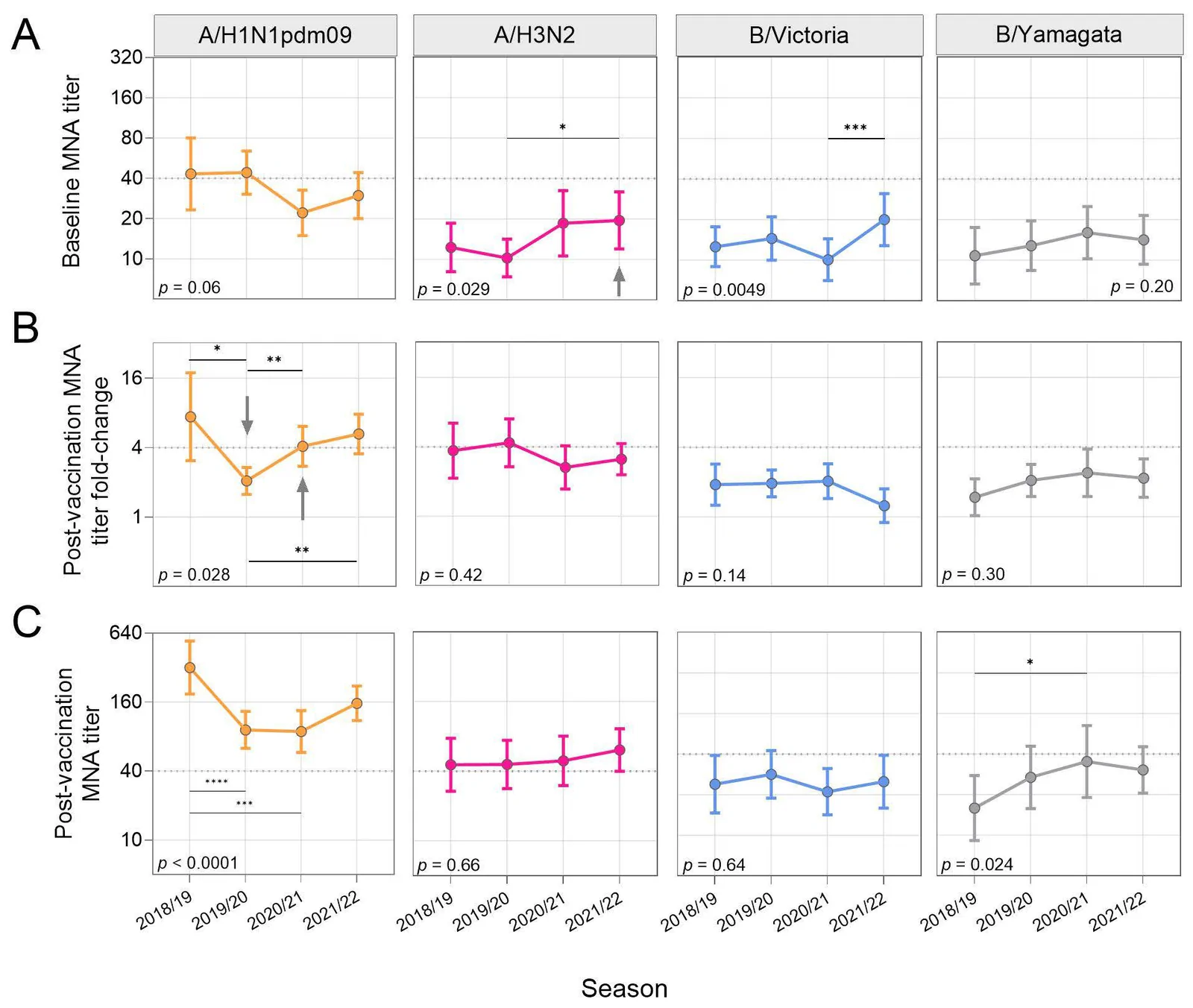
Pre- and post-vaccination neutralizing antibody titers during four influenza epidemic seasons. (**A**) Pre-vaccination baseline antibody titers measured on day 1; (**B**) fold changes in post-vaccination antibody titers measured on day 21 relative to baseline (day 1); and (**C**) post-vaccination antibody titers measured on day 21. GMTs with 95% CIs are shown. Antibodies were measured against the corresponding vaccine viruses. The dotted line indicates the seroprotection threshold in (**A**,**C**) and the biologically significant titer fold increase in (**B**). Results of the mixed-effects model are shown in the lower-right corner of each graph (*p* values for the season factor). Statistically significant differences between seasons are indicated by asterisks: * *p* < 0.05, ** *p* < 0.01, *** *p* < 0.001 and **** *p* < 0.0001, based on Holm–Sidak multiple-comparison tests. Individual data are presented in Supplementary Figure S3.

Neutralizing antibody titers showed patterns that differed from those of HAI antibody titers, depending on the vaccine component (Figure A1, Supplementary Figure S2). For A/H1N1pdm09, both HAI and MNA showed a statistically significant decline in post-vaccination antibody fold-increases during the second season and an overall downward trend in post-vaccination GMTs. However, the decline in post-vaccination MNA titers was more pronounced and occurred earlier (a sharp decline was already evident in the second season), indicating that the neutralizing response was more sensitive to high baseline titers.

For A/H3N2, the assays showed discrepant patterns: baseline HAI titers increased 5.4 4-fold over four seasons (*p* < 0.001), whereas MNA titers showed a less pronounced trend (-maximum increase, 1.9-fold; *p* = 0.029). This finding may indicate that the accumulation of hemagglutinating antibodies was not accompanied by a proportional increase in neutralizing activity, possibly because of annual changes in the antigenic properties of the H3N2 strain.

For the B-lineage vaccine components (Victoria and Yamagata), vaccine components, substantial differences between the two methods were observed, except a significant increase in post-vaccination titers to B/Yamagata in the 2020/21 season that was detected by both assays (2.9-fold by HAI and 2.3-fold by MNA).

Thus, the MNAs revealed an earlier and more pronounced decline in the neutralizing response to A/H1N1pdm09 after booster vaccination, whereas the HAI assay was more sensitive to the cumulative increase in antibodies to A/H3N2 and to the introduction of the B/Yamagata strain in the quadrivalent vaccine.

**Figure 2:**
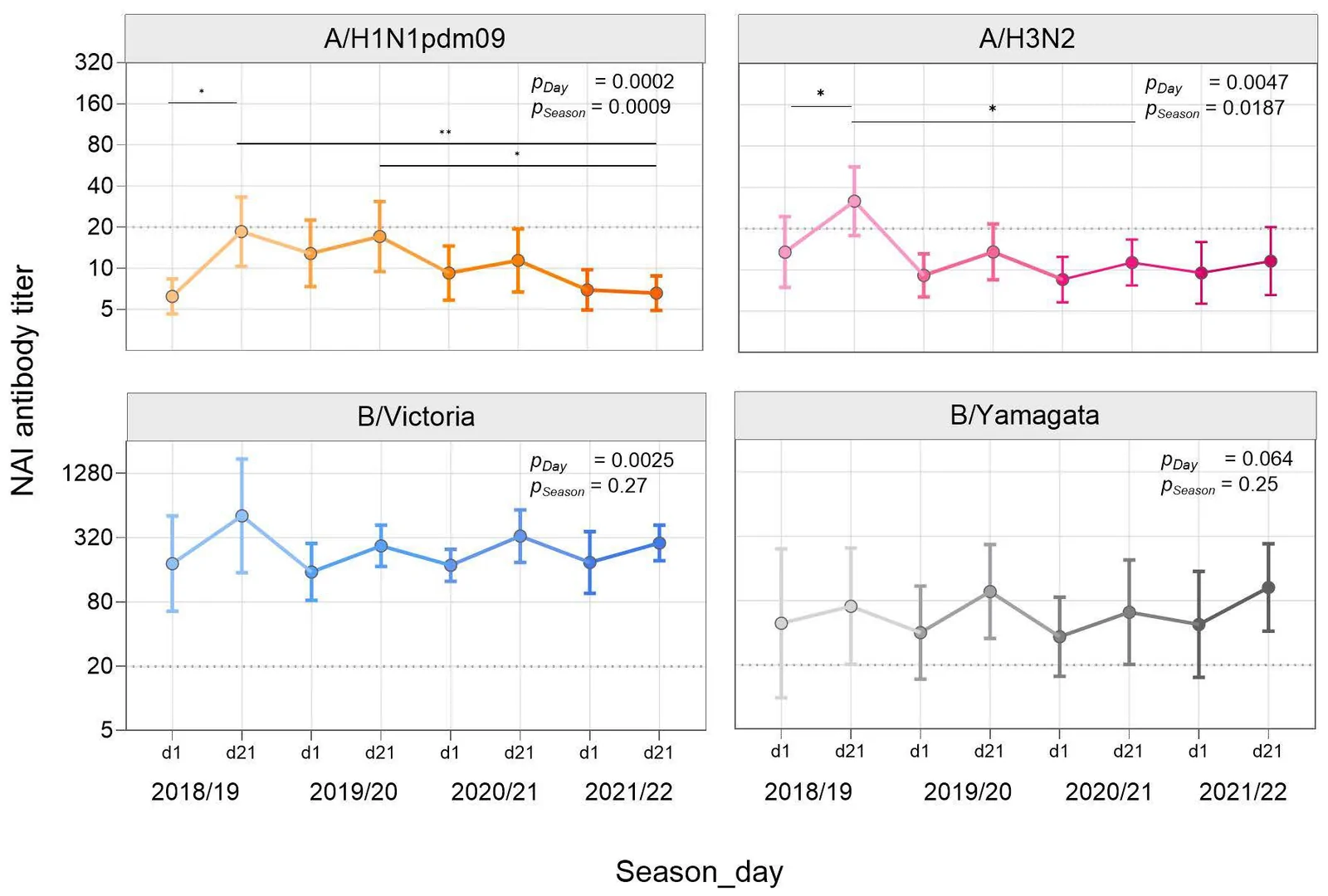
Pre- and post-vaccination neuraminidase-inhibiting antibody titers during four influenza epidemic seasons. GMTs with 95% CIs are shown. Day 1, pre-vaccination; day 21, post-vaccination. Results of the mixed-effects model are shown in the upper-right corner of each graph (*p* values for the day and season factors). Statistically significant changes based on Holm–Sidak multiple-comparison tests are indicated by asterisks: * *p* < 0.05, and ** *p* < 0.01. Thedotted line indicates the biologically significant threshold. Individual data are presented in Supplementary Figure S3.

The antibody response to neuraminidase, the second influenza antigen in the vaccines, showed less pronounced dynamics (Figure 2, Supplementary Figure S3). A statistically significant 3.1-fold increase in NAI antibody titers to N1 was observed between days 1 and 21 after the first vaccination (2018/19 season). No significant peaks were observed after repeated vaccination in the subsequent periods (2020/21 and 2021/22), and post-vaccination titers decreased significantly in the final season (2021/22). A similar pattern was observed for N2 neuraminidase: only the first immunization produced a significant 2.6-fold increase in NAI antibodies, and a 2.8-fold decrease in post-vaccination titers was observed in the third season. NAI antibody titers to B/Victoria were consistently high (range, 128–512). Small increases in titers (1.6-to 1.9- fold) were observed between the pre- and post- vaccination time points (*p* = 0.0025); however, pairwise comparisons within individual seasons were not statistically significant. Responses to B/Yamagata showed the greatest variability in antibody reactivity to NA, and no statistically significant changes were observed.

**Figure 3:**
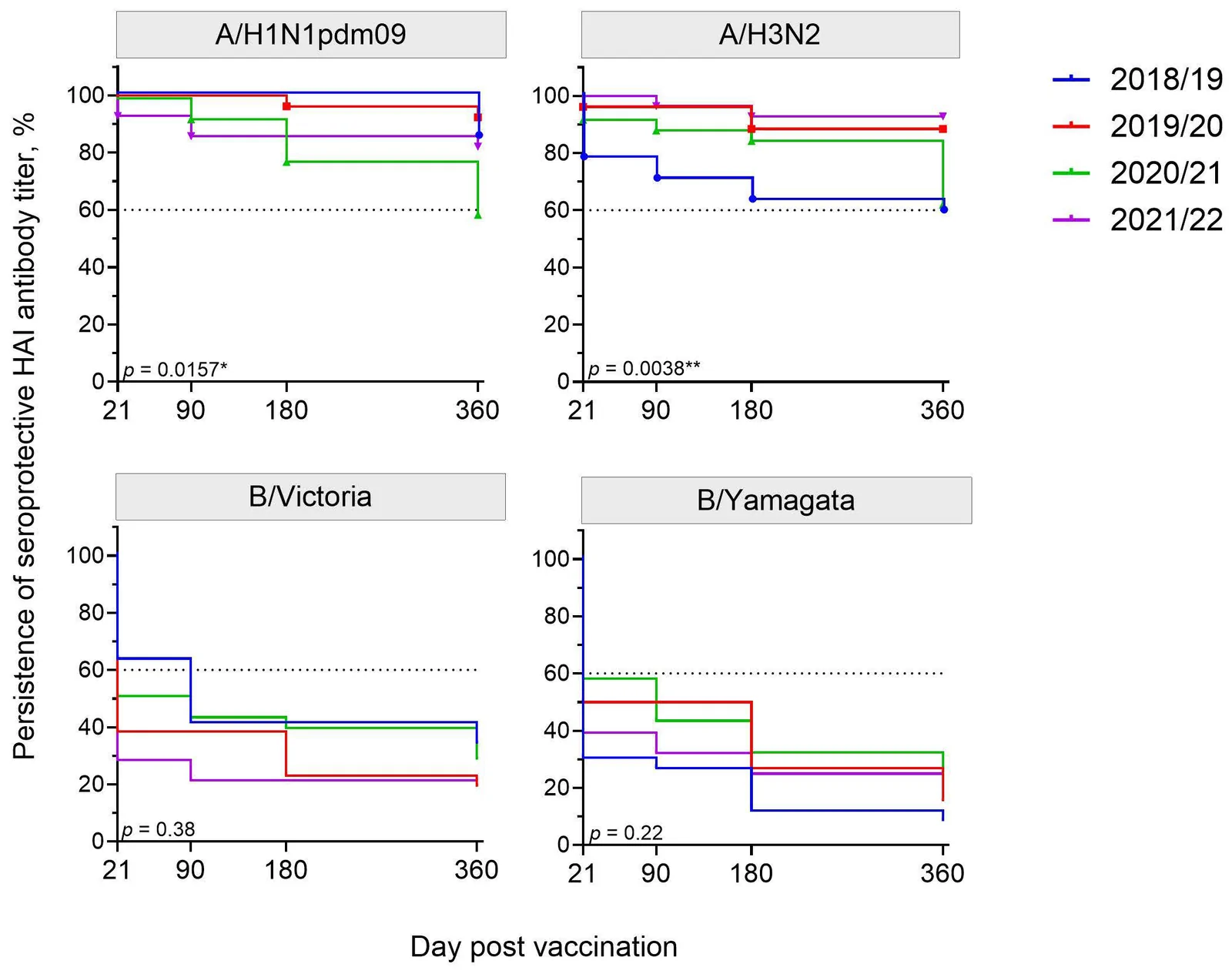
Persistence of seroprotective HAI titers in vaccinated participants (percentage of participants HAI titers ≥ 40). The dotted line indicates the 60% seroprotection rate threshold, as one of the CPMP criteria for influenza vaccine immunogenicity [28]. Statistically significant changes based on Mantel-Cox test are indicated by asterisks: * *p* < 0.05, ** *p* < 0.01.

**Figure 4:**
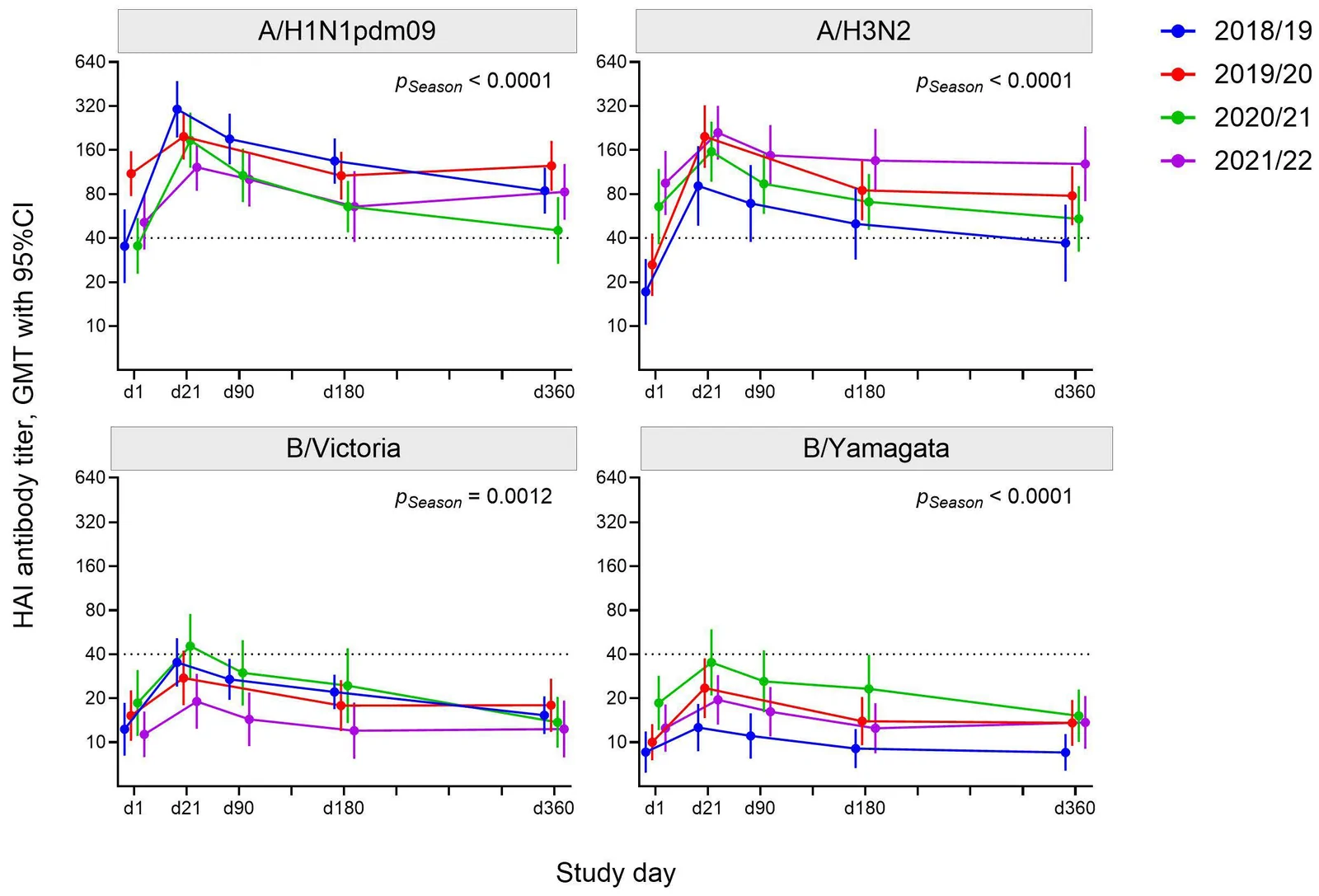
Dynamics of geometric mean HAI antibody titers in the study cohort during the four-year observation period. Error bars indicate 95% CIs. Results of the mixed-effects model are shown in the upper-right corner of each graph (*p* values for the season factor). The main statistically significant differences between seasons at days 1 and 360, based on post hoc Holm–Sidak multiple-comparison tests, were as follows: A/H1N1pdm09, *p* < 0.01 for 2019/20 vs. 2020/21 and 2019/20 vs. 2021/22; A/H3N2, *p* < 0.0001 for 2018/19 vs. 2021/22; B/Victoria, *p* < 0.01 for 2020/21 vs. 2021/22; and B/Yamagata, *p* < 0.0001 for 2018/19 vs. 2020/21. The dotted line indicates the seroprotection threshold.

Finally, we compared the duration of the HAI antibody response among seasons. At each scheduled visit, participants were classified according to whether their HAI titer remained at or above the seroprotection threshold. Continued persistence at or above this threshold was treated as “survival”. For each participant, the first scheduled day on which the titer fell below 40 was defined as the event time (e.g., day 180, event = 1). Participants with titers at or above 40 on day 360 were censored (day = 360, event = 0). The resulting data for each season were plotted as Kaplan–Meier curves, and seasons were compared using the Mantel–Cox test. This approach enabled comparison among seasons while accounting for seroprotection rates at multiple time points. (Figure 3). The duration of the HAI antibody response to the A/H1N1pdm09 component was longest during the first vaccination season (2018/19) and decreased significantly in the 2020/21 season; the seroprotection rate on day 360 was 79% in 2018/19 versus 50% in 2020/21 (Figure S4). Conversely, the duration of the response to A/H3N2 increased over time, from lowest duration after the first vaccination in 2018/19 to its longest duration after the fourth vaccination in 2021/22 season. The corresponding seroprotection rates on day 360 were 57% and 61%, respectively. The duration of the antibody response to influenza B components remained short and did not change significantly over the four years. Parallel analysis of GMT trajectories confirmed the same interseasonal differences in responses to the A/H1N1pdm09 and A/H3N2 components. The analysis also revealed a significant increase in the response to the B/Yamagata component in 2020/21, accompanied by a peak response to B/Victoria that subsequently declined significantly in 2021/22 (Figure 4).

## 4. Discussion

Longitudinal monitoring of post-vaccination humoral immunity to influenza over four years revealed complex relationships among pre-existing antibody titers, vaccine strain updates, and HA- and NA-specific responses to individual influenza virus subtypes.

For the A/H1N1 component, we observed the classic inverse relationship between pre-vaccination antibody titers and post-vaccination fold increase. This finding is consistent with the phenomena of “antigenic seniority” and antibody-mediated restriction. A probable underlying mechanism is the rapid clearance of vaccine antigens by high baseline titers of neutralizing antibody, which limits restimulation and expansion of peripheral memory B-cell populations [9,34]. Moreover, the MNAs revealed an earlier and sharper decline in neutralizing titers, suggesting a more rapid decline in functional immunity in the presence of high baseline antibody titers, whereas HAI antibodies persisted longer.

In contrast, the A/H3N2 component showed a pronounced cumulative trend. Over the four-year observation period, pre-vaccination antibody titers increased significantly and consistently, reaching a maximum in the fourth year. This repeated antigen exposure and the resulting accumulation of baseline antibodies were accompanied by a sharp decline and subsequent stabilization of fold increases at low levels during the third and fourth years. The highest seroprotection rate and longest antibody response duration for A/H3N2 were observed in the final 2021/22 season. This pattern may be related to the greater antigenic divergence of the A/H3N2 vaccine component, which was updated annually.

The B/Victoria component showed relatively stable dynamics across all four years, with a modest increase through the 2020/21 season. The only exception was a statistically significant decrease in B/Victoria antibody titers during the 2021/22 season, which coincided with the absence of an update to the vaccine strain for this lineage. The relatively weak response to the B/Victoria component compared with the responses to influenza A components is consistent with previous reports of lower or more variable vaccine-induced HAI responses to influenza B viruses [35,36,37]. The weaker response to influenza B may have obscured patterns that were apparent for the influenza A components, such as the inverse relationship between baseline levels and post-vaccination fold increases.

Conversely, the response to the B/Yamagata component coincided with the transition to quadrivalent vaccines. During the first year of the study, when trivalent vaccines lacking the B/Yamagata lineage dominated, baseline titers remained low, and virtually no increase in antibodies was observed. The introduction of QIVs was associated with statistically significant increases in post-vaccination HAI titers in the 2019/20 and 2020/21 seasons withsignificant changes in MNA titers in 2020/21. The subsequent downward trend in the fourth season (2021/22) and the absence of significant changes in antibody fold increases may have resulted from repeated vaccination with the same vaccine strains. Analysis of the response in 2021/22 was less likely to be confounded by natural exposure because the B/Yamagata lineage had disappeared from global circulation [38,39]. Thus, the antibody trajectory primarily reflected the response to repeated vaccination with the same strain. The later observation periods (the 2020/21 and 2021/22 seasons) coincided with the COVID-19 pandemic, during which unprecedented non-pharmaceutical interventions, such as lockdowns, universal masking, and travel restrictions—were implemented globally. These measures substantially suppressed seasonal influenza circulation, and induced a severe evolutionary bottleneck [40], affecting all influenza strains but having a particularly pronounced effect on the B/Yamagata lineage [41]. During the final season of our study, B/Yamagata was still included in the quadrivalent formulations used in the cohort. Subsequent global evidence supported removing this lineage and returning to trivalent formulations, an approach endorsed by the WHO for the 2024/25 season [42].

Although assessments of influenza vaccine efficacy have historically relied on HAI assays, NAI antibody titers provide an important, but often overlooked layer of protection. Compared with HA antigen, NA undergoes antigenic drift more slowly, resulting in broader antibody cross-reactivity within the same subtype and, in some cases, across subtypes [43,44]. Even when the HA-directed response is limited by high baseline immunity or low viral circulation, a robust, cross-reactive NAI response may inhibit viral shedding, reduce transmission, and mitigate disease severity [45]. In this study, the response to NA was generally weaker than that to HA, which may reflect the lower and non-standardized NA content of split-virion or subunit inactivated vaccines, together with HA immunodominance and possible steric hindrance [46,47,48,49]. Statistically significant increases in NAI antibody titers to N1 and N2 were observed only during the 2018/19 season, with no significant peaks in subsequent periods. Moreover, during the 2021/2022 season, the increased use of split vaccines—which have previously been shown to elicit stronger immune responses to NA than subunit vaccines [26]—did not reverse this trend. Nevertheless, vaccine formulation alone cannot explain these changes because NA content and functional activity are not standardized and may vary among manufacturers, seasons, and vaccine lots [50]. It may also be related to the use of the same antigenically outdated NA strain to assess responses over four years, which is a limitation of the present study. Other proposed mechanisms underlying reduced immunogenicity include antigen clearance by preexisting antibodies [8,9].

Most studies of antibody persistence evaluate GMTs and their relative changes long after vaccination compared with pre-vaccination levels [10,17,51]. Here, we suggested an alternative approach based not on relative GMTs, but on the proportion of vaccinees with antibody titers at or above the seroprotection threshold (≥40). An HAI titer of ≥40 is a conventional immunological threshold that originated from human challenge studies of individual protection [52,53] and was subsequently evaluated at the population level in epidemiological studies [54,55,56]. Comparison among influenza seasons revealed an association between vaccine strain changes and longer persistence of antibody titers above the seroprotective threshold for A/H3N2 and B/Victoria vaccine components.

We acknowledge that the limited demographic diversity of the study cohort restricts the generalizability of our findings. Nevertheless, the small cohort enabled longitudinal monitoring at the individual level and statistical comparisons among seasons using repeated-measures data. Another important consideration is natural influenza exposure, which may influence the magnitude and duration of vaccine responses [57,58]. The strong and persistent A/H1N1pdm09-specific response during the 2018/19 season may have been associated with exposure to the A/H1N1pdm09 virus circulating at that time. However, a similar pattern was not observed for A/H3N2, which circulated during the same period. We also observed no increase in the duration of B/Victoria-specific antibody persistence in 2019/20 despite the predominance of influenza B viruses during that season. Furthermore, no influenza infections were detected among vaccinated participants during the four-year observation period. We therefore consider the influence of natural exposure on longitudinal antibody persistence in this cohort to have been limited. We suggest that the main determinants were the interplay between repeated vaccination and changes in vaccine strains.

## 5. Conclusions

Post-vaccination humoral immunity to influenza A subtypes was influenced by the cohort’s immunological history. High pre-vaccination antibody titers were associated with significant reductions in relative post-vaccination fold increases; however, post-vaccination antibody titers and seroprotection rates were not adversely affected. The transition to quadrivalent vaccine formulations beginning in the 2019/20 season was associated with a statistically significant increase in baseline antibody titers against the B/Yamagata lineage and an overall enhancement of the response to influenza B components. Annual updates to vaccine strains likely contributed to the long duration of antibody responses to the A/H3N2 and B/Victoria vaccine components over four seasons. Regular updates to vaccine strains may therefore enhance the persistence of HA-targeted humoral immune responses.

## Figures and Tables

**Table 1:** Vaccine composition recommended for the Northern Hemisphere throughout the study. Changes in vaccine strains are highlighted in bold.

Component	Season 2018–2019	Season 2019–2020	Season 2020–2021	Season 2021–2022
A/H1N1pdm09	A/Michigan/45/2015	**A/Brisbane/02/2018**	**A/Guangdong-Maonan/SWL1536/2019**	**A/Victoria/2570/2019**
A/H3N2	A/Singapore/ INFIMIH-16-0019/2016	**A/Kansas/14/2017**	**A/Hong Kong/2671/2019**	**A/Cambodia/e0826360/2020**
B/Victoria	**B/Colorado/06/2017**	B/Colorado/06/2017	**B/Washington/02/2019**	B/Washington/02/2019
B/Yamagata	B/Phuket/3073/2013 *	B/Phuket/3073/2013 *	B/Phuket/3073/2013 *	B/Phuket/3073/2013 *

*—Applies only to quadrivalent formulations.

**Table 2:** Characteristics of the cohort at the begining of the four-year study.

Characteristics	Value
Total number of volunteers	N = 28
Age	
Median (IQR)	55 (37–61)
Min–Max	18–76
Sex, n (%)	
Female	21 (75%)
Male	7 (25%)
Ethnicity, n (%)	
White	28 (100%)
Smoking, n (%)	
Yes	8 (28.6%)
No	16 (57.1%)
Unknown	4 (14.3%)
Chronic diseases, n (%)	
No	10 (35.7%)
Cardiovascular disease	9 (32.1%)
COPD	1 (3.6%)
Bronchial asthma	1 (3.6%)
Diabetes	1 (3.6%)
Autoimmune disease	2 (7.1%)
Rheumatological disease	3 (10.7%)
Neuromuscular disease	0
Immunodeficiency/transplantation	0
Renal disease	0
Obesity	0
Tuberculosis	0
HIV	0
Other	9 (32%)
Vaccination in the previous season 2017/18	
Yes	11 (39.3%)
No	17 (60.7%)
Vaccination in the previous 3-years	
Yes	16 (57.1%)
No/Unknown	12 (42.9%)

**Table 3:** Types of inactivated influenza vaccines used throughout the study.

Season	Formulation			
	Split	Subunit	Trivalent	Quadrivalent
2018/19	54%	46%	100%	0%
2019/20	61%	39%	46%	54%
2020/21	57%	43%	50%	50%
2021/22	89%	11%	18%	82%

## Data Availability

The data presented in this study are available from the corresponding author upon reasonable request. The data are not publicly available due to the institution’s policy.

## Supplementary Materials

The following supporting information can be downloaded at https://www.mdpi.com/article/10.3390/vaccines14090800/s1, Figure S1: Pre- and post-vaccination hemagglutination inhibition antibody titers during four influenza epidemic seasons (individual dynamics); Figure S2: Pre- and post-vaccination neutralizing antibody titers during four influenza epidemic seasons (individual dynamics); Figure S3: Pre- and post-vaccination neuraminidase inhibiting antibody titer during four influenza epidemic seasons (individual dynamics); Figure S4: Seroprotection rate in the study cohort at scheduled time points during four-year observation period; Figure S5: Follow-up rates of the studied cohort at five visits during four epidemic seasons.

## Abbreviations

The following abbreviations are used in this manuscript:

WHO	World Health Organization
TIV	Trivalent Influenza Vaccine
QIV	Quadrivalent Influenza Vaccine
HA	Hemagglutinin
HAI	Hemagglutination Inhibition
MNA	Microneutralization Assay
NAI	Neuraminidase Inhibition
ELISA	Enzyme-linked Immunosorbent Assay
ELLA	Enzyme-Linked Lectin Assay
A/H1N1pdm09	A/H1N1pdm09 vaccine component
A/H3N2	A/H3N2 vaccine component
B/Victoria	B/Victoria-lineage vaccine component
B/Yamagata	B/Yamagata-lineage vaccine component
GMT	Geometric Mean Titer
95% CI	95% confidence interval
CPMP	European Committee on Patented Medicines CPMP/BWP/214/1996

## Appendix A

**Table A1:** Influenza and COVID-19 incidence in the study cohort during the four-year observation period.

Cases of Infection	Season			
	2018/19	2019/20	2020/21	2021/22
Symptomatic COVID-19, PCR-confirmed, n (%)	-	1 (3.5%)	5 (17.9%)	3 (10.7%)
Symptomatic influenza, PCR-confirmed, n (%)	-	-	-	-
Asymptomatic influenza, suspected *, n (%)				
A/H1N1pdm09	-	-	-	-
A/H3N2	-	-	-	-
B/Victoria	-	-	1 (3.5%)	-
B/Yamagata	-	-	-	-
**Dominant influenza strain(s)** during the epidemic season **	A/H3N2 = A/H1N1pdm09	B/Victoria >> A/H1N1pdm09	B/Victoria (several cases)	A/H3N2 >> B/Victoria

* Suspected influenza infection was defined as a ≥4-fold increase in antibody titer between days 90 and 180 or between days 180 and 360, confirmed by both HAI and MNA. ** Data are specific to the Russian Federation and are based on references [32,33].
