# Supplementary material for: Duration of the Humoral Immune Response to Influenza Vaccination in a Cohort of Repeatedly Vaccinated Adults over Four Consecutive Epidemic Seasons: 2018/19–2021/22 Observational Study
Source: Vaccines (Basel). 2026 Sep 11;14(9):800. doi: 10.3390/vaccines14090800 (PMC13611785; doi:10.3390/vaccines14090800)
Supplement: Supplementary file 1 [file vaccines-14-00800-s001.zip › vaccines-4475711-supplementary.pdf]

## Supplementary Material

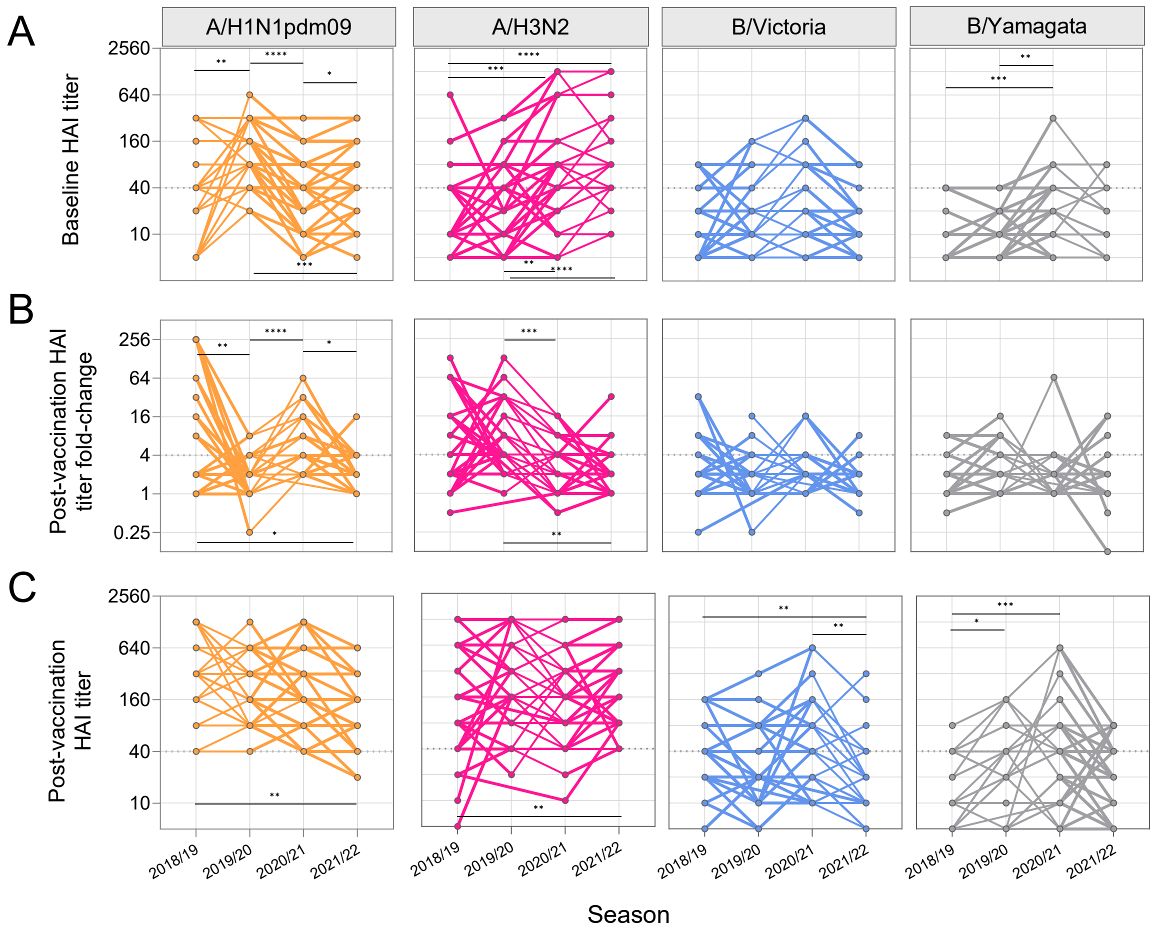


***Figure S1.*** Pre- and post-vaccination hemagglutination-inhibition antibody titers during four influenza epidemic seasons (individual trajectories). Statistically significant differences based on Holm–Sidak multiple-comparison tests are indicated by asterisks: * $p < 0.05$, ** $p < 0.01$, *** $p < 0.001$, and **** $p < 0.0001$ (see Figure 1 for details). The dotted line indicates the seroprotection threshold in (a) and (c) and the biologically significant titer fold increase in (b).

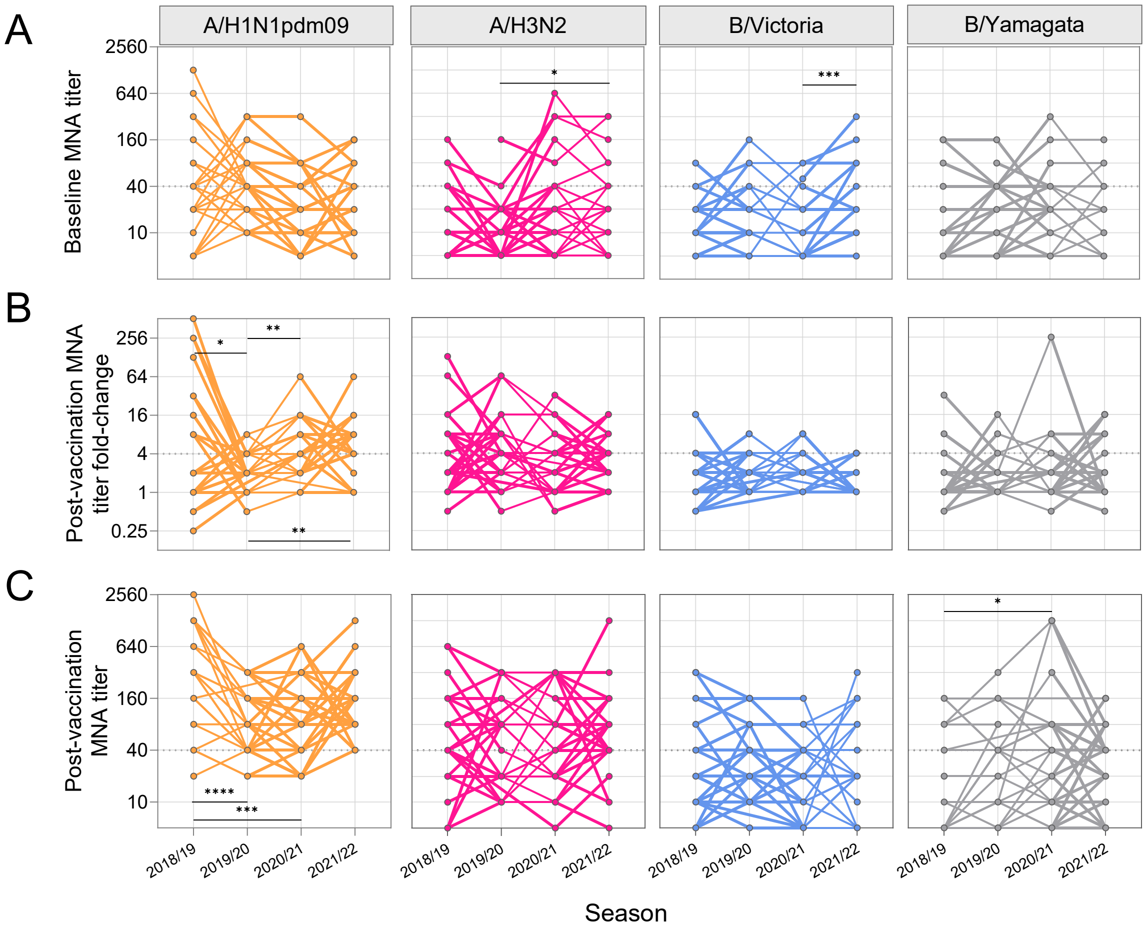


***Figure S2.*** Pre- and post-vaccination neutralizing antibody titers during four influenza epidemic seasons (individual trajectories). Statistically significant differences based on Holm–Sidak multiple-comparison tests are indicated by asterisks: * $p < 0.05$, ** $p < 0.01$, *** $p < 0.001$, and **** $p < 0.0001$ (see Figure B1 for details). The dotted line indicates the seroprotection threshold in (a) and (c) and the biologically significant titer fold increase in (b).

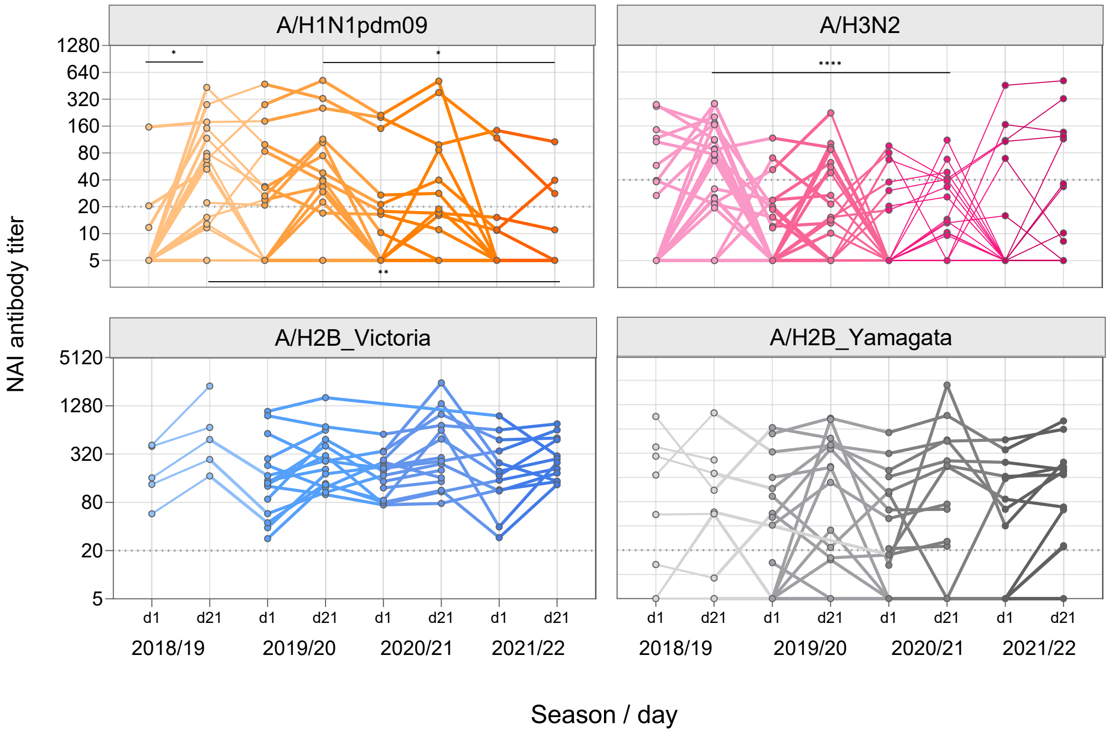


***Figure S3.*** Pre- and post-vaccination neuraminidase-inhibiting antibody titers during four influenza epidemic seasons (individual trajectories). Day 1, pre-vaccination; day 21, post-vaccination. Statistically significant differences based on Holm–Sidak multiple-comparison tests are indicated by asterisks: * $p < 0.05$, ** $p < 0.01$, and **** $p < 0.0001$ (see Figure 2 for details).

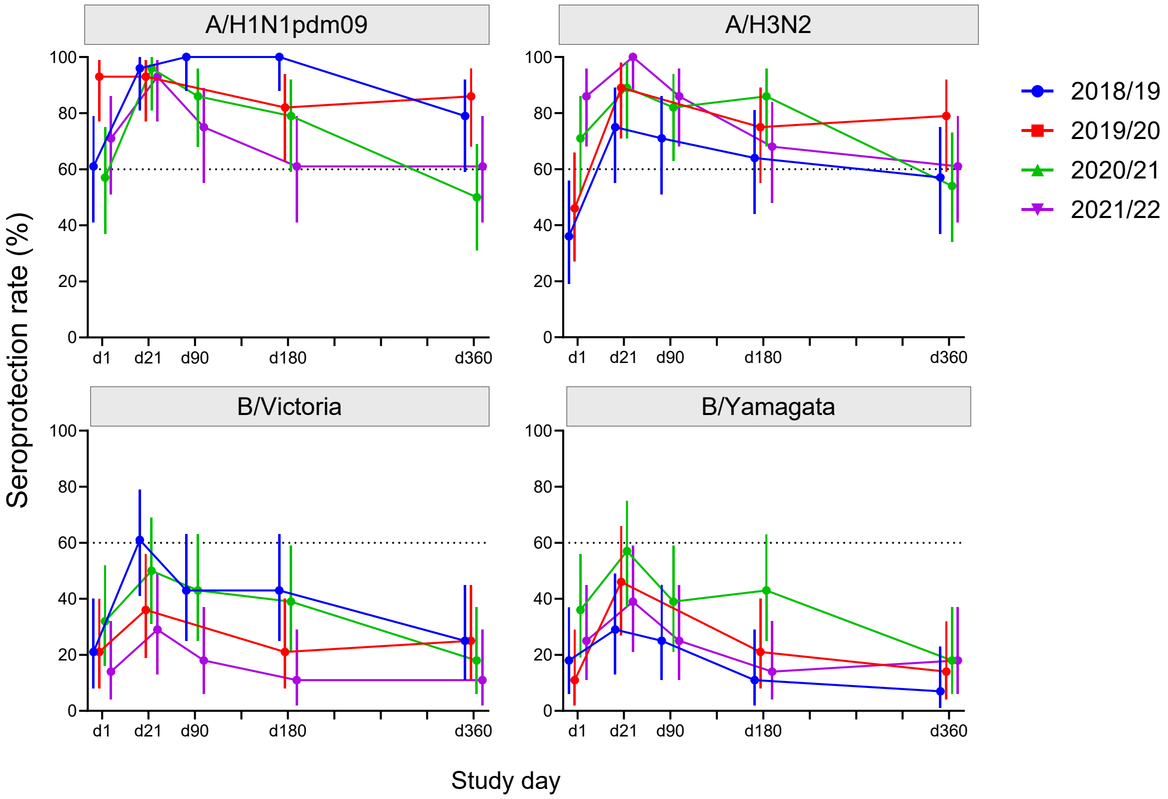


***Figure S4.*** Seroprotection rates in the study cohort at scheduled time points during the four-year observation period. The percentage of participants with HAI titers ≥ 40 at each time point in each season is shown; error bars indicate 95% CIs.

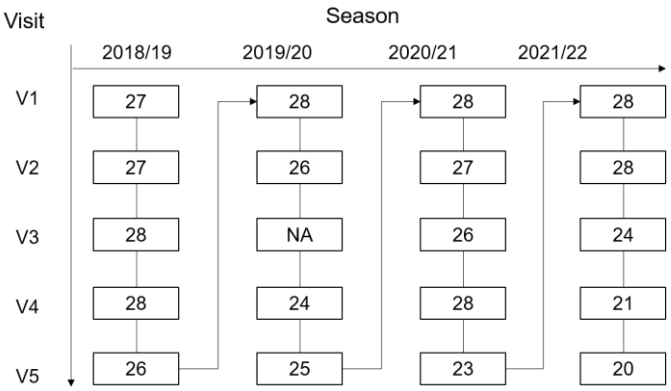


***Figure S5.*** Follow-up rates in the study cohort at five visits during four epidemic seasons. Numbers indicate the serum samples available for analysis at each time point. NA, samples not available for the specified time point.
